# Araloside C attenuates atherosclerosis by modulating macrophage polarization via Sirt1-mediated autophagy

**DOI:** 10.18632/aging.102708

**Published:** 2020-01-27

**Authors:** Yun Luo, Shan Lu, Ye Gao, Ke Yang, Daoshun Wu, Xudong Xu, Guibo Sun, Xiaobo Sun

**Affiliations:** 1 Institute of Medicinal Plant Development, Peking Union Medical College and Chinese Academy of Medical Sciences, Beijing 100193, China; 2 Beijing Key Laboratory of Innovative Drug Discovery of Traditional Chinese Medicine (Natural Medicine) and Translational Medicine, Beijing 100193, China; 3 Key Laboratory of Bioactive Substances and Resource Utilization of Chinese Herbal Medicine, Ministry of Education, Beijing 100193, China; 4 Key Laboratory of Efficacy Evaluation of Chinese Medicine Against Glyeolipid Metabolism Disorder Disease, State Administration of Traditional Chinese Medicine, Beijing 100193, China; 5 Key Laboratory of New Drug Discovery Based on Classic Chinese Medicine Prescription, Chinese Academy of Medical Sciences, Beijing 100193, China; 6 College of Pharmacy, Harbin University of Commerce, Harbin 150076, Heilongjiang, China; 7 Collaborative Innovation Center of Yangtze River Delta Region Green Pharmaceuticals, Zhejiang University of Technology, Hangzhou 310014, Zhejiang, China

**Keywords:** atherosclerosis, macrophage polarization, autophagy, Sirt1, Araloside C

## Abstract

Atherosclerosis-related cardiovascular disease is still the predominant cause of death worldwide. Araloside C (AsC), a natural saponin, exerts extensive anti-inflammatory properties. In this study, we explored the protective effects and mechanism of AsC on macrophage polarization in atherosclerosis in vivo and in vitro. Using a high-fat diet (HFD)-fed ApoE-/- mouse model and RAW264.7 macrophages exposed to ox-LDL, AsC was evaluated for its effects on polarization and autophagy. AsC significantly reduced the plaque area in atherosclerotic mice and lipid accumulation in ox-LDL-treated macrophages, promoted M2 phenotype macrophage polarization, increased the number of autophagosomes and modulated the expression of autophagy-related proteins. Moreover, the autophagy inhibitor 3-methyladenine and BECN1 siRNA obviously abolished the antiatherosclerotic and M2 macrophage polarization effects of AsC. Mechanistically, AsC targeted Sirt1and increased its expression, and this increase in expression was associated with increased autophagy and M2 phenotype polarization. In contrast, the effects of AsC were markedly blocked by EX527 and Sirt1 siRNA. Altogether, AsC attenuates foam cell formation and lessens atherosclerosis by modulating macrophage polarization via Sirt1-mediated autophagy.

## INTRODUCTION

Cardiovascular disease (CVD) is still the leading cause of death worldwide due to its high morbidity and mortality; it not only reduces human life span but also places a heavy burden to the national health care system according to the latest authoritative statistics [1]. Atherosclerosis, a major inducer of CVD, is a chronic inflammatory disease arising from an imbalance in lipid metabolism and a maladaptive immune response driven by the accumulation of cholesterol-laden macrophages in the arterial wall [2]. During atherosclerotic lesion formation, macrophage polarization, which leads to diverse phenotypes, is a critical process that depends on various stimuli [3]. Notably, it has been concluded that the anti-inflammatory M2 macrophage phenotype exerts atheroprotective effects, although anti-inflammatory CD163^+^ macrophages also promote angiogenesis and vascular permeability [4]. Oxidized low-density lipoprotein (ox-LDL), which contributes directly to macrophage polarization, induces foam cell formation, ultimately promoting plaque formation [5]. Previous opinions on atherosclerosis therapy have mainly focused on lipid-lowering, antithrombotic, antioxidative and anti-inflammatory strategies, and have ignored macrophage polarization [6]. Thus, the pharmacological targeting of macrophage polarization represents a promising therapeutic strategy for atherosclerosis.

Increasing literature has reported the significant role of autophagy, a lysosome-mediated conserved cellular pathway that controls protein and organelle degradation in human health and disease [7]. As expected, autophagy is an emerging therapeutic target for atherosclerosis that has been summarized recently [8, 9]. According to the recent literature, macrophage autophagy induction prevents atherosclerosis [10], whereas impaired macrophage autophagy increases atherosclerotic plaque formation [11]. Silent information regulator 1 (Sirt1), a member of the sirtuin class of proteins, is widely studied and shows atheroprotective effects in macrophages [12], endothelial cells [13], and vascular smooth muscle cells [14]. Thus, screening for potent selective Sirt1 activators has been the focus of research in the antiatherosclerosis drug development field, and more research into the mechanism by which Sirt1 activation affects atherosclerosis is imperative.

**Figure 1:**
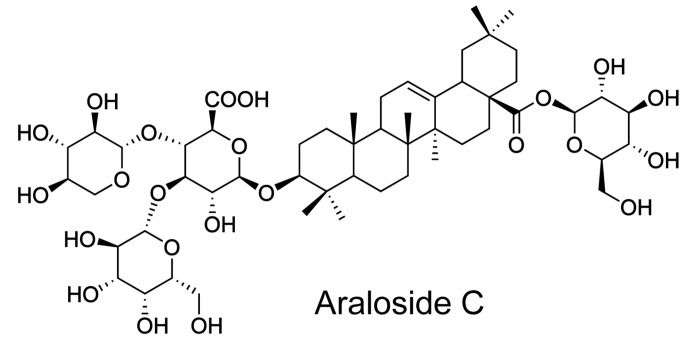
**The chemical structure of Araloside C (AsC).**

Clinically, although lipid-lowering statins and anti-inflammatory canakinumab have been used for atherosclerosis treatment, they also have inevitable side effects [15–17] and do not meet clinical needs. In recent decades, strategies involving natural products have focused on cell autophagy for atherosclerosis prophylaxis and treatment, and many natural compounds have been indicated to exhibit excellent antiatherosclerotic properties [18]. Araloside C (AsC, Figure 1), a bioactive triterpenoid, is the major active constituent of *Aralia elata* (Miq.) Seem, which has been widely used in traditional Chinese medicine [19]. Recently, our group reported that AsC alleviated hypoxia/reoxygenation-induced cardiomyocyte apoptosis *in vitro* [20] and *ex vivo* [21] studies. Moreover, we found that total saponins of *Aralia elata* (Miq.) (TASAES) protected against endothelial cell injury and atherosclerosis in ApoE-/- mice [22, 23]. According to the emerging reports of the cardioprotective effects of AsC and the endothelial protective effects of TASAES, we believe that the antiatherosclerotic effects of AsC and its possible molecular mechanism need to be elucidated.

Based on our previous research, this study is the first to investigate the antiatherosclerotic effects and underlying mechanism of AsC on ox-LDL-induced foam cell formation. Additionally, we speculate that AsC attenuates foam cell formation and lessens atherosclerosis by modulating macrophage polarization via Sirt1-mediated autophagy.

## RESULTS

### AsC attenuated atherosclerosis in HFD-induced ApoE-/- mice and reduced foam cell formation *in vitro*

**Figure 2:**
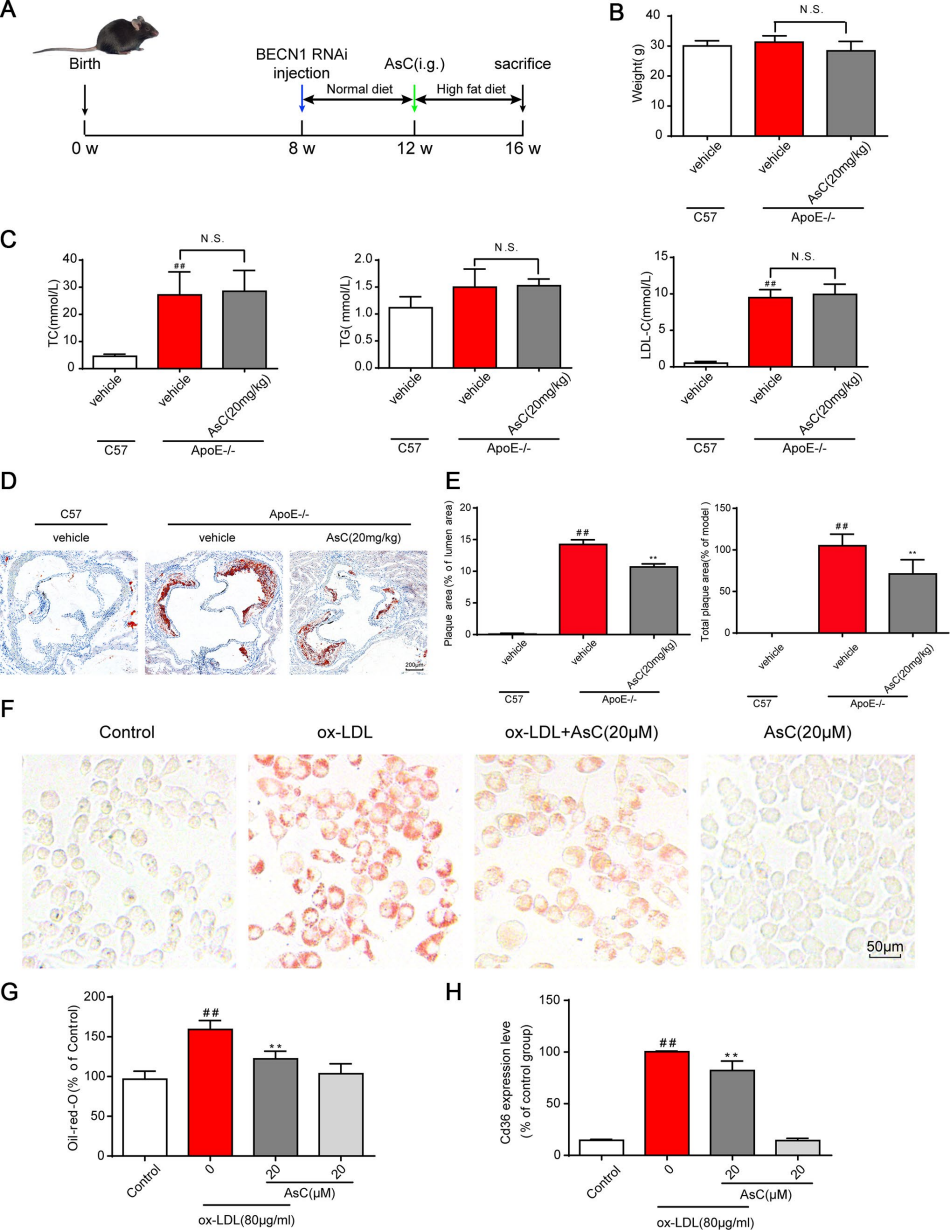
**AsC attenuated atherosclerosis in HFD-fed ApoE-/- mice and reduced foam cell formation *in vitro*.** All mice were fed a HFD in the presence or absence of AsC (20 mg·kg^-1^·day^-1^, i.g.) for 4 weeks. In the *in vitro* assay, RAW264.7 cells were pretreated with AsC (20 μM) for 12 h, and then exposed to ox-LDL for another 24 h. (**A**) Experimental protocol of the *in vivo* study. (**B**) Body weight. (**C**) Blood lipid levels. (**D**) Representative images of oil red O staining of the aortic root. (**E**) Quantification of the plaque area by oil red O staining. (**F**) Representative images of oil red O staining in ox-LDL-treated RAW264.7 cells. (**G**) Quantification of oil red O staining, as detected by a microplate reader. (**H**) Cd36 expression level in ox-LDL-treated RAW264.7 cells, as determined by flow cytometry. The data are presented as the means ± SDs (n = 5). ^##^*P* < 0.01 *vs.* the control group, ^**^P < 0.01 *vs.* the model group; N.S. means no significance.

To test whether AsC exerts an antiatherosclerotic effect, we first measured the weight, blood lipid levels and atherosclerotic area at the aortic root of high-fat diet (HFD)-treated ApoE-/- mice according to our previous method [24]. Unexpectedly, no significant differences in weight, blood lipid levels (Figure 2B and 2C), fat and lean proportion, or necrotic core (Supplementary Figure 1) were observed upon AsC treatment. Moreover, as shown in Figure 2D and 2E, the mice in the AsC group developed significantly smaller plaque areas in the aortic root than those in the model group after 4 weeks of treatment. We further examined the effects of AsC on serum lipid profiles. These data suggest that the antiatherosclerotic effect of AsC was not dependent on lipid level regulation.

Macrophage-derived foam cells play an important role in atherosclerosis formation. Next, we analyzed the effects of AsC on ox-LDL-induced foam cells *in vitro*, and the results showed that AsC remarkably decreased foam cell formation (Figure 2F and 2G). Meanwhile, compared with ox-LDL group, it ameliorated Cd36 expression (Figure 2H, Supplementary Figure 2), which strengthened our conclusion. Collectively, these findings confirm that AsC had antiatherosclerotic effects and reduced foam cell formation.

### AsC polarized macrophages to an M2-like phenotype

Mounting evidence points to a key role of macrophage polarization in plaque progression and vulnerability [5].

**Figure 3:**
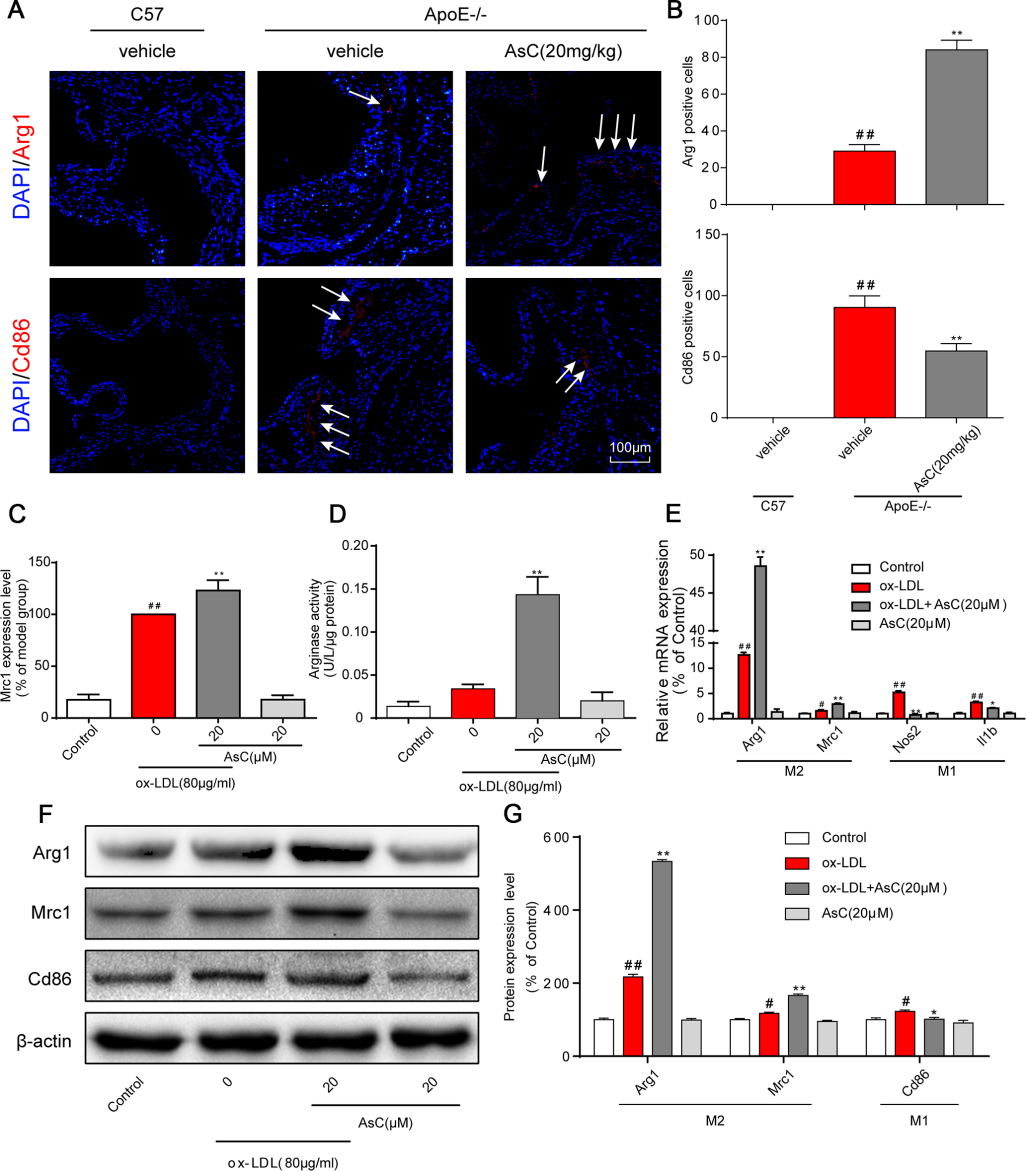
**AsC polarized macrophages to an M2-like phenotype.** All mice were fed a HFD in the presence or absence of AsC (20 mg·kg^-1^·day^-1^, i.g.) for 4 weeks. In the *in vitro* assay, RAW264.7 cells were pretreated with AsC (20 μM) for 12 h, and then exposed to ox-LDL for another 24 h. (**A**) Dual immunofluorescence staining for Arg1 (red) or Cd86 (red) and DAPI (blue) in lesions in the aortic root. (**B**) Quantification of the relative fluorescence intensity. (**C**) The Mrc1 expression level in ox-LDL-treated macrophages, as determined by flow cytometry. (**D**) Arginase activity was measured as described in the Methods section. (**E**) mRNA levels of Arg1, Mrc1, Nos2 and Il1b in macrophages, as quantified by real-time PCR. (**F**) Representative photographs of Mrc1, Cd86 and Arg1 expression in ox-LDL-treated macrophages, as evaluated by western blot analysis. (**G**) Statistical results of Mrc1, Cd86 and Arg1 expression levels compared with those in the control group. The data are presented as the means ± SDs (n = 5). ^#^*P* < 0.05, ^##^*P* < 0.01 *vs.* the control group, ^**^P < 0.01 *vs.* the model group.

We thus detected the expression of macrophage polarization markers in the aortic root in control and AsC-treated mice. Immunostaining analysis showed that the expression of Cd86 was significantly decreased in the AsC group, whereas the expression of Arg1 was significantly increased (Figure 3A and 3B). A similar tendency was observed in the ox-LDL-induced macrophage model by flow cytometry analysis (Figure 3C, Supplementary Figure 3). Arginase activity is also a macrophage polarization marker, as indicated in Figure 3D. AsC treatment obviously increased arginase activity. Moreover, we also detected the mRNA and protein expression levels of M1 and M2 macrophage markers. As shown in Figure 3E–3G, AsC significantly downregulated Nos2, Il1b, and Cd86 expression, and upregulated Arg1 and Mrc1 expression, which was consistent with a previous study [25]. These results indicate that AsC pretreatment was able to polarize macrophages to an M2-like phenotype.

### AsC induced macrophage autophagy

**Figure 4:**
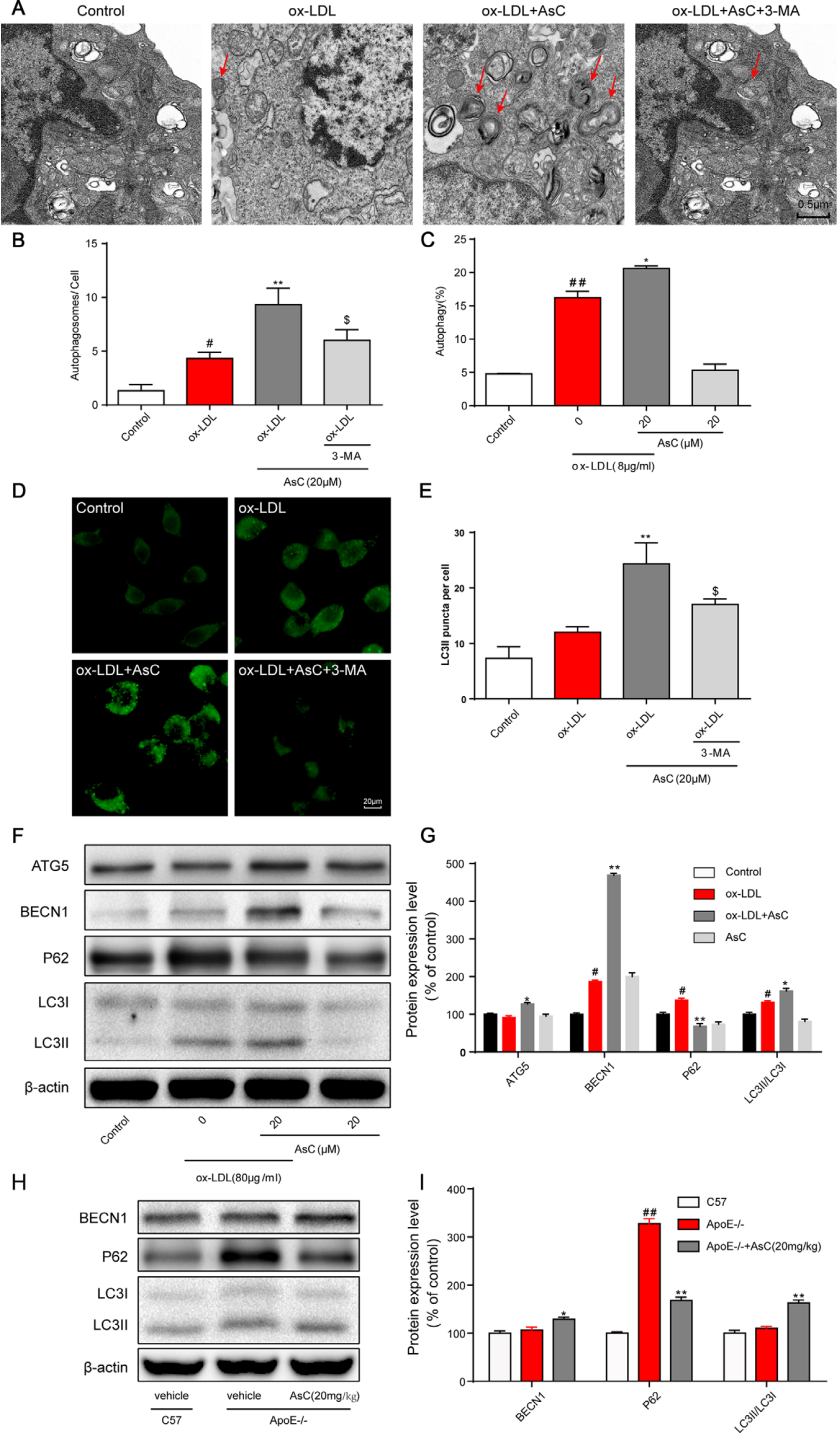
**AsC induced macrophage autophagy. RAW264.7 cells were pretreated with 3-MA (5 mM) for 2 h, treated with AsC (20 μM) for 12 h, and then exposed to ox-LDL for another 24 h.** (**A**) Representative photographs of autophagosomes (red arrows) examined using a JEOL JEM1230 electron microscope. (**B**) Statistical results of autophagosomes. (**C**) Summarized data showing the percentage of cells that were positive for CytoID fluorescence, as detected by flow cytometry analysis. (**D**) Representative photographs of LC3II staining. (**E**) Statistical results of LC3II-positive cells. (**F**) Representative photographs of ATG5, BECN1, P62, LC3 and β-actin expression in ox-LDL-treated macrophages, as evaluated by western blot analysis. (**G**) Statistical results of ATG5, BECN1, P62, LC3II/LC3I expression levels compared with those in the control group. (**H**) Representative photographs of BECN1, P62, LC3 and β-actin expression in aortic lysates. (**I**) Statistical results of BECN1, P62, LC3II/LC3I expression levels compared to those in the control group. The data are presented as the means ± SDs (n = 5). ^#^*P* < 0.05, ^##^*P* < 0.01 *vs.* the control group, ^*^P < 0.05, ^**^P < 0.01 *vs.* the model group; ^$^*P* < 0.05 *vs.* the ox-LDL and AsC group.

Ongoing laboratory studies have demonstrated that autophagy is a therapeutic target for atherosclerosis [8]. To determine whether AsC regulates autophagy, we first investigated autophagosomes by TEM, the most valid method for both qualitative and quantitative analysis of autophagy [26]. The results showed that AsC pretreatment increased the number of autophagosomes in ox-LDL-treated macrophages, but that the number of autophagosomes decreased when the cells were pretreated with the autophagy inhibitor 3-MA (Figure 4A and 4B). Cyto-ID® and flow cytometric assays demonstrated that AsC treatment increased autophagic vacuoles and flux (Figure 4C), further confirming AsC-induced autophagy in ox-LDL-stimulated macrophages. Next, we determined the level of LC3II, one of the gold standard markers of autophagosome formation [27]. Our data indicated that AsC dramatically elevated LC3II expression levels, suggesting that autophagic flux was increased, and these levels were also blocked by 3-MA (Figure 4D and 4E). To further confirm the role of AsC in the modulation of autophagic flux, we measured the expression levels of autophagy-related proteins. As shown in Figure 4F and 4G, AsC significantly increased the ratio of LC3II/LC3I, which is considered an accurate indicator of autophagy, upregulated BECN1 and ATG5 expression levels, and reduced the P62 expression level. Similar results were confirmed in aortic lysates (Figure 4H and 4I). Taken together, these findings strongly indicate that pretreatment with AsC enhanced ox-LDL-induced macrophage autophagy level.

### Autophagy inhibition blocked AsC-mediated antiatherosclerotic effects and macrophage polarization

**Figure 5:**
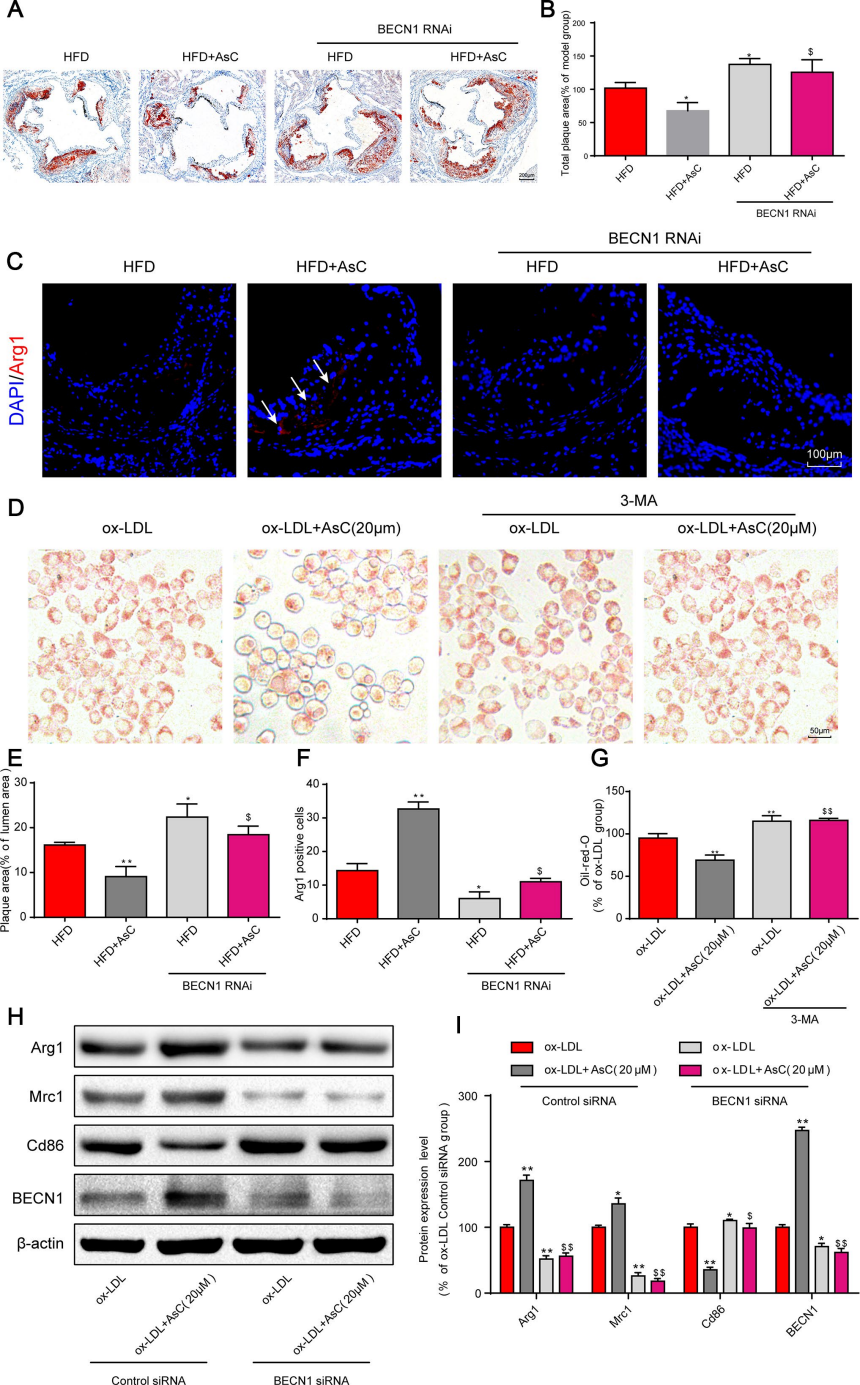
**Autophagy inhibition abolished AsC-mediated antiatherosclerotic effects and macrophage polarization.** All mice were fed a HFD in the presence or absence of AsC (20 mg·kg^-1^·day^-1^, i.g.) for 4 weeks. In the *in vitro* assay, RAW264.7 cells were pretreated with 3-MA (5 mM) for 2 h, treated with AsC (20 μM) for 12 h, and then exposed to ox-LDL for another 24 h. (**A**) Representative images of oil red O staining of the aortic root. (**B**) Quantification of the total plaque area. (**C**) Dual immunofluorescence staining forArg1 (red) and DAPI (blue) in lesions in the aortic root. (**D**) Representative images of oil red O staining of ox-LDL-treated RAW264.7 cells. (**E**) The percentage of plaque area relative to lumen area. (**F**) Quantification of relative fluorescence intensity. (**G**) Quantification of oil red O staining, as detected by a microplate reader. (**H**) Representative photographs of Arg1, Mrc1, Cd86 and BECN1 expression, as evaluated by western blot analysis. (**I**) Statistical results of Mrc1, Cd86 and Arg1 expression levels compared with those in the ox-LDL-treated group. The data are presented as the means ± SDs (n = 5). ^*^P < 0.05, ^**^P < 0.01 *vs.* the model group; ^$^*P* < 0.05, ^$$^*P* < 0.01 *vs.* the ox-LDL and AsC group.

Based on our above research results, we next investigated the effects of AsC-mediated autophagy on atherosclerosis and macrophage polarization. First, our *in vivo* data showed that the reduction in plaque area by AsC was significantly reversed by the autophagy inhibition (Figure 5A and 5D). Similarly, the increase in Arg1 expression by AsC was significantly inhibited by BECN1 knockdown (Figure 5B and 5E). Moreover, the inhibitory effect of AsC on ox-LDL-induced foam cell formation was also abrogated by 3-MA in RAW264.7 cells (Figure 5C and 5F). In addition, AsC-mediated macrophage polarization was also abolished by BECN1 siRNA *in vitro* (Figure 5G and 5H). Together, these data suggest that AsC mitigated atherosclerosis and promoted macrophage polarization through the promotion of macrophage autophagy.

### AsC promoted Sirt1 expression in macrophages

**Figure 6:**
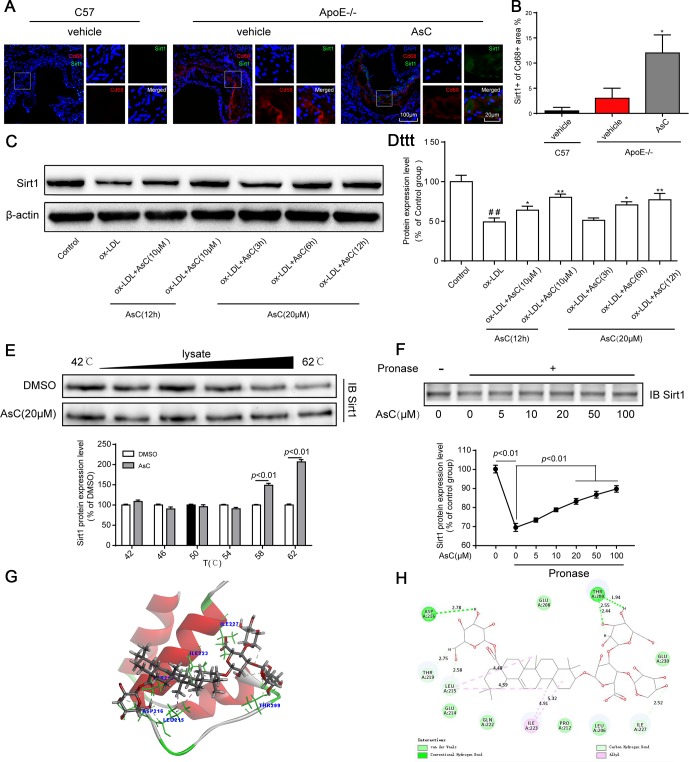
**AsC promoted Sirt1 expression in macrophages.** All mice were fed a HFD in the presence or absence of AsC (20 mg·kg^-1^·day^-1^, i.g.) for 4 weeks. In the *in vitro* assay, RAW264.7 cells were pretreated with AsC (20 μM) for 12 h, and then exposed to ox-LDL for another 24 h. (**A**) Aortic roots from ApoE−/− mice were stained for the macrophage marker Cd68 and coprobed with antibodies against Sirt1. (**B**) Quantification of Sirt1 expression in aortic root lesions. (**C**) Representative photographs of Sirt1 expression, as evaluated by western blot analysis. (**D**) Statistical results of the Sirt1 expression level compared with that in the control group. (**E**) Cellular thermal shift assay (CETSA) using macrophage lysates, which were exposed to AsC (20 μM). (**F**) AsC promoted the resistance of its target protein Sirt1 to proteases (DARTS). (**G**) Three-dimensional modeling of the binding of AsC to the binding domain of Sirt1. (**H**) Two-dimensional ligand interaction diagram of AsC and SIRT1. The data are presented as the means ± SDs (n = 5). ^##^*P* < 0.01 *vs*. the control group; ^*^P < 0.05, ^**^P < 0.01 *vs*. the model group.

Next, we explored the mechanism of AsC-mediated autophagy in macrophages. Sirt1 is a well-known autophagy regulator [28]; therefore, we determined the Sirt1 expression level in macrophages in the aortic root and ox-LDL-activated macrophages. Immunofluorescence colocalization assays indicated that compared with vehicle, AsC notably increased Sirt1 expression in atherosclerotic macrophages (Figure 6A and 6B). Moreover, our results showed that Sirt1 levels increased in response to AsC pretreatment in a dose- and time-dependent manner (Figure 6C and 6D).

Meanwhile, we explored the interaction between AsC and Sirt1 by CETSA and a DARTS assay. As shown in Figure 6E and 6F, compared with the control, treatment with AsC significantly inhibited Sirt1 degradation induced by temperature and pronase, which was in accordance with a previous study [29]. Next, we further examined the potential interaction between AsC and Sirt1 protein through molecular docking. As shown in Figure 6G, the N-terminus of Sirt1 forms an independently folded three α-helix bundle, and AsC binds to the helix-turn-helix motif. Meanwhile, it was observed that AsC interacted extensively with several important amino acids in the N-terminus: it formed hydrogen bonds with THR 209, LEU 215, ASP 216, THR 219 and ILE 227, van der Waals interactions with LEU 206, GLU 208, PRO 212, GLU 214, GLN 222 and GLU 230, and alkyl interactions with ILE 223 (Figure 6H). This result is basically the same as that reported by other researchers [30].

The *in vivo*, *in vitro* and molecular docking results, suggest that AsC prevented foam cell and atherosclerosis formation by targeting and upregulating Sirt1 expression.

### AsC-mediated polarization and autophagy in ox-LDL-treated macrophages were Sirt1-dependent

**Figure 7:**
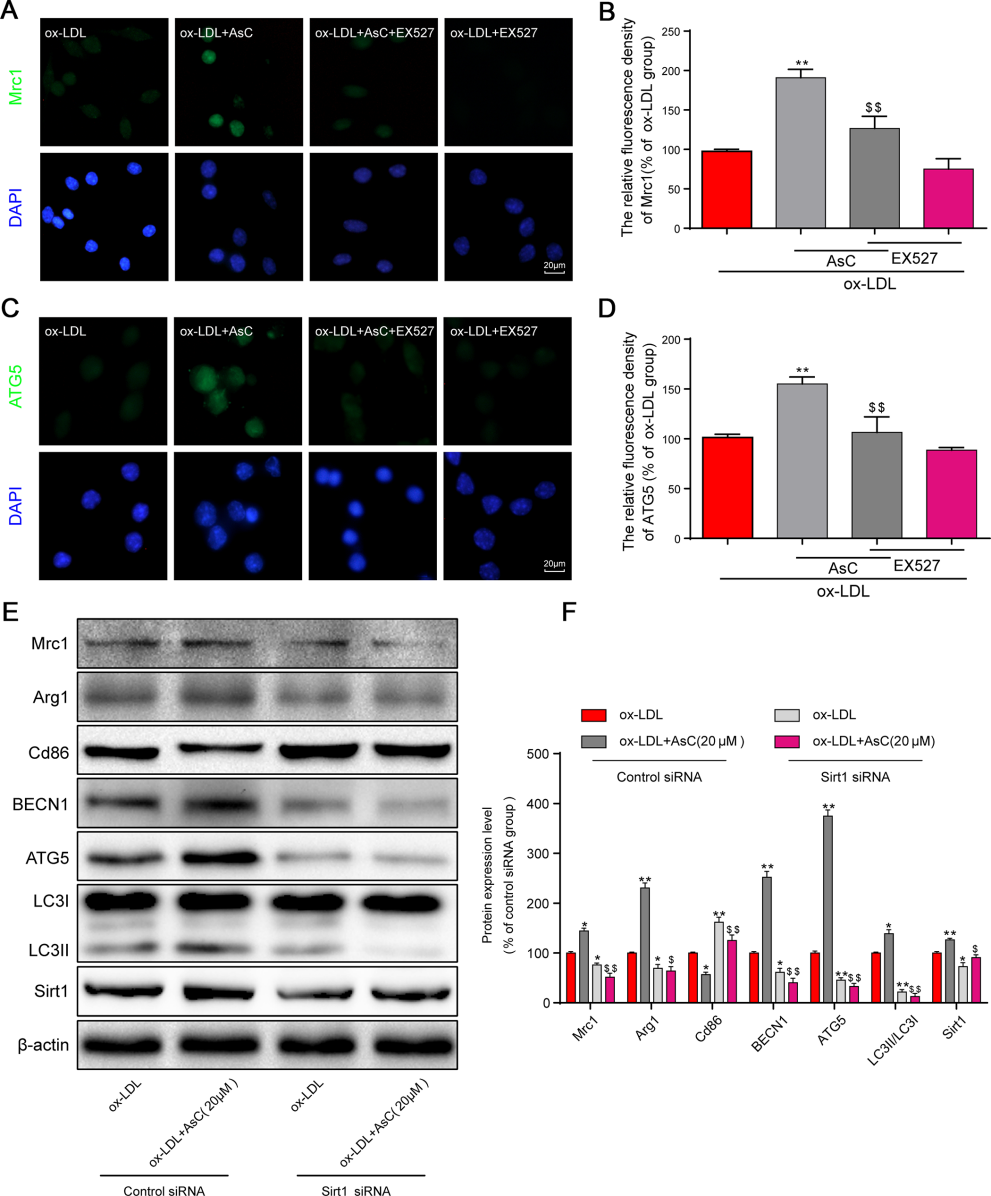
**AsC-induced autophagy and M2 phenotype polarization were Sirt1-dependent in ox-LDL-treated macrophages. Sirt1 was inhibited by 10 μM EX527 for 6 h or knocked down by siRNA, as described in the Materials and Methods section.** Twenty-four hours posttransfection, cells were treated with AsC (20 μM) for 12 h and then incubated with ox-LDL (80 μg/mL) for an additional 24 h. (**A**, **C**) Representative immunofluorescence images showing Mrc1 and ATG5 expression in RAW264.7 cells. (**B**, **D**) The relative quantitative analysis of Mrc1 and ATG5 fluorescence in RAW264.7 cells. (**E**) Representative western blot analysis of Mrc1, Arg1, Cd86, BECN1, ATG5, LC3, Sirt1 and β-actin in macrophages. (**F**) Quantification of the expression of Mrc1, Arg1, Cd86, BECN1, ATG5, LC3, and Sirt1. The data are presented as the means ± SDs (n = 5). ^*^P < 0.05, ^**^P < 0.01 *vs.* the ox-LDL group; ^$^*P* < 0.05, ^$$^*P* < 0.01 *vs.* the ox-LDL and AsC group.

Previous studies have shown that Sirt1 activation is necessary for autophagic flux activation [31]. It has been demonstrated that Sirt1 could be an ideal target for drugs [32]. We hypothesized that Sirt1 is involved in autophagic flux activation by AsC. Thus, the Sirt1 inhibitor EX527 and Sirt1 siRNA were utilized to investigate whether Sirt1 activation was essential for AsC-induced autophagic flux in our study. After treatment with EX527, the modulation of Mrc1 and ATG5 by AsC was not detected in macrophages, demonstrating that the activation of polarization and autophagy were abolished by Sirt1 inhibition (Figure 7A–7D). Sirt1 siRNA had similar effects, and these results collectively demonstrated that AsC enhanced autophagy through SIRT1 signaling (Figure 7E and 7F).

**Figure 8:**
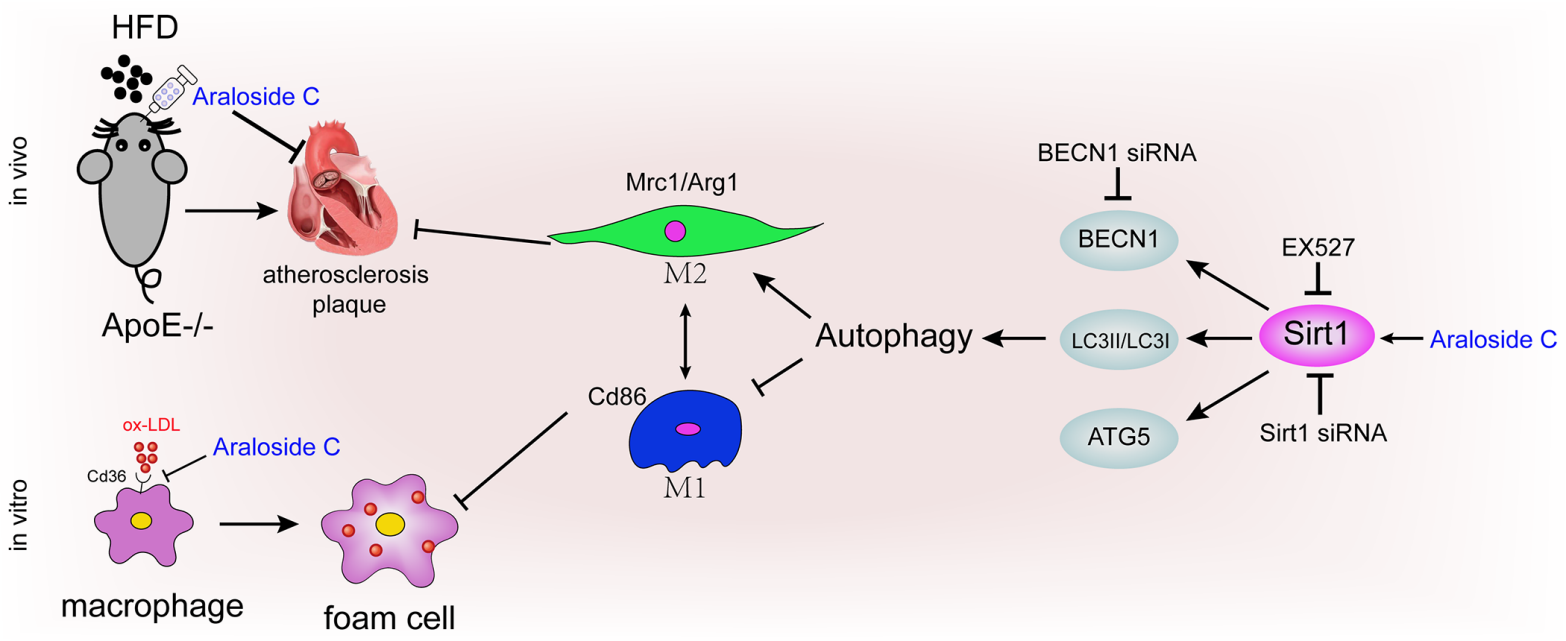
**Diagram of the proposed molecular mechanism of the antiatherosclerotic effect of AsC.**

Together, these results suggest that decreased Sirt1 expression is involved in the impairment of autophagy in macrophages and that the effects of AsC in promoting autophagy may occur through Sirt1 activation (Figure 8).

## DISCUSSION

Although atherosclerosis has been effectively treated through lipid-reducing and anti-inflammatory drugs, the adverse effects associated with muscle symptoms and glucose homeostasis [33] compelled us to find effective and less toxic drugs to improve patient prognosis. In this study, we demonstrated that AsC, a naturally derived saponin, exerted a potent antiatherosclerotic effect. We further confirmed the beneficial pharmacological effect of AsC on macrophage autophagy and phenotype switching. Mechanistically, the administration of AsC potently targeted and activated Sirt1 signaling, which subsequently mediated macrophage autophagy and polarization.

Recently, many natural products have been studied to determine their impact on atherosclerosis [18]. Our group and several others have reported that TASAES exerts therapeutic effects on myocardial damage [34, 35] and atherosclerosis [22, 23]. AsC, isolated from TASAES, was also reported to have powerful cardioprotective effects against ischemia-reperfusion injury in our previous study [20, 21]. Moreover, we demonstrated that another AsC analogue can protect against ox-LDL-damaged endothelial cell injury via autophagy induction [36], which prompted us to explore whether AsC has antiatherosclerotic effects and determine its protective mechanism. Expectedly, our data demonstrated that AsC ameliorated plaque area in atherosclerosis in HFD-fed ApoE-/- mice. Foam cell formation is a key event in the development of atherosclerotic plaques. Furthermore, we found that AsC reduced ox-LDL-induced foam cell formation *in vitro* through classical oil red O staining and Cd36 detection. In addition, AsC had no obvious effects on blood lipid levels, body weight or the fat or lean to body weight ratio, which implied that the antiatherosclerotic effects of AsC are different from statins. The above results led us to understand how AsC executed its antiatherosclerosis ability.

In past decades, research on antiatherosclerosis drugs mainly focused on lipid-reducing, antiinflammatory, and antioxidative strategies, endothelial protection and foam cell inhibition. Few studies have focused on macrophage polarization, which is also a promising target for atherosclerosis therapy [37, 38]. Generally, the anti-inflammatory macrophage phenotype is considered atheroprotective, although CD163^+^ macrophages promote atherosclerosis [3, 4]. In this study, we observed a notable change in macrophage polarization. AsC treatment elevated Arg1 expression and reduced Cd86 expression in atherosclerosis, suggesting phenotypic switch to anti-inflammatory M2 macrophages. These results in addition to our oil red O staining results, revealed that the AsC-mediated polarization of macrophages to the M2 was correlated with the dynamics of atherosclerotic plaque regression. Furthermore, the changes in gene or protein expression of macrophages Arg1, Mrc1, Cd86, Nos2 and Il1b were in accordance with the *in vivo* results. Taken together, our results indicate that AsC specifically regulated macrophage polarization both *in vitro* and *in vivo*.

It has been widely reported that macrophage autophagy can reduce the accumulation of foam cells and inhibit the formation and development of plaques [39, 40]. Here, we have analyzed autophagic flux in macrophages. Our results showed that AsC significantly increased the number of macrophage autophagosomes, which were blocked by 3-MA. Meanwhile, the immunofluorescence data showed that the increase in LC3II-positive macrophages by AsC was dramatically decreased by 3-MA. Similar results were also observed at the protein level in ox-LDL-treated macrophages. These results suggest that AsC observably promoted macrophage autophagy.

There is increasing evidence indicating that autophagy is important for the induction of M2 macrophages. Many natural products have been found to mediate the crosstalk of polarization and autophagy in macrophages for disease therapy [41–43]. To study the relationship between ability of AsC to increase autophagy and the macrophage polarization, we constructed BECN1 RNAi mice for use in ApoE-/- mice [44] and BECN1 siRNA for use in macrophages. In the current study, we found that the decrease in plaque area and increase in Arg1 expression by AsC were markedly inhibited by BECN1 RNAi. Moreover, AsC-mediated foam cell inhibition and macrophage phenotype switching were also abolished by 3-MA and BECN1 siRNA, respectively. Therefore, these data strongly support that AsC can enhance autophagy, which contributes to M2 phenotype polarization.

Next, we investigated the molecular mechanism by which AsC protects against atherosclerosis. Sirt1, a nicotinamide adenine dinucleotide-dependent protein deacetylase, has gained attention for its protective effects against atherosclerosis [45, 46]. Excitingly, Sirt1 can modulate macrophage polarization [47] and autophagy [48].

Our present results showed that compared with vehicle, AsC increased the expression of Sirt1 *in vivo* and *in vitro*. Moreover, AsC treatment elevates the temperature and enzyme stability of Sirt1, which indicated that Sirt1 may be a target of AsC. Molecular docking results confirmed this conclusion. To confirm the crucial roles of Sirt1 in AsC-mediated macrophage autophagy and polarization, a Sirt1 inhibitor and siRNA were used to inhibit Sirt1 expression. Mrc1 and ATG5 expression were sharply abrogated by EX527 in macrophages. Subsequently, Sirt1 siRNA reversed the AsC induced alterations in Mrc1, Arg1 and Cd86 expression and inhibited the AsC-induced increase in LC3-II/LC3-I, BECN1 and ATG5 expression, and the reduction in p62 expression. Consistent with previous studies, the present results showed that the upregulation of Sirt1 by AsC promoted cell autophagy [28] and polarization [42]. Along with the *in vitro, in vivo* and molecular docking results, these data led us to hypothesize that AsC modulates autophagy and polarization in macrophages by targeting Sirt1 and upregulating its expression.

In conclusion, our findings in this paper provide novel insights into the molecular mechanisms of AsC against atherosclerosis (Figure 8). AsC should be developed as an efficient candidate, and macrophage polarization should also be considered as a drug target in the clinic. However, in the present work, we selected Sirt1 as the only target gene for the study. However, the antiatherosclerotic effects of AsC are intricate, and one gene cannot fully explain the actions of the compound. Multiple targets, multiple signaling pathways, and multiple biological processes should be explored in future studies to fully elucidate the molecular mechanisms of AsC against atherosclerosis.

## MATERIALS AND METHODS

### Reagents

Ox-LDL (by copper ion-induced LDL oxidation, MDA=35 nM) was obtained from Union-Bio Technology (Beijing, China). AsC was isolated in our previous study (Wang et al., 2014) at the Institute of Medicinal Plant Development (Beijing, China). Dimethylsulfoxide (DMSO), 3-(4,5-dimethylthiazol-2yl-)-2, oil red O, DAPI and 3-methyladenine (3-MA) were purchased from Sigma-Aldrich (St. Louis, MO, USA). The DMEM Basal Medium and fetal calf serum (FBS) were obtained from HyClone (Logan, UT, USA). The CytoID Autophagy Detection Kit was obtained from Enzo Life Sciences (Farmingdale, NY, USA). Cd36 and Mrc1 flow cytometry antibodies were purchased from Biolegend (San Diego, CA, USA). EX527 was obtained from Target Molecule Corp. (Shanghai, China). Antibodies against P62, LC3II and pronase were obtained from Sigma-Aldrich (St. Louis, MO, USA). NP40 lysis buffer was obtained from Beyotime Biotechnology (Shanghai, China). Anti-mouse and anti-rat IgG (H+L) F(ab')2 fragment antibodies were acquired from Cell Signaling Technology (Danvers, MA, USA). Antibodies against Sirt1, Cd86, Mrc1, Arg1, and BECN1 were purchased from Abcam (Cambridge, United Kingdom). All other antibodies were purchased from Santa Cruz Biotechnology (Santa Cruz, CA, USA).

### Animals

All animal experiments were approved by the Institutional Animal Care and Use Committee (IACUC) at the Chinese Academy of Medical Sciences and Peking Union Medical College, Beijing, China. Six-week-old (17 ± 1 g) male ApoE-/- mice with a C57BL/6 N background were purchased from Beijing Vital River Laboratory Animal Technology Co., Ltd. (Beijing, China) and maintained in conventional cages in a temperature controlled facility (temperature: 22 ± 1°C; humidity: 60%) with a 14 h light/10 h dark cycle. A BECN1 knockdown mouse model was constructed by injecting a lentivirus (Lv) expressing RNA inference (RNAi) targeting BECN1, which was purchased from Shanghai GeneChem Co. Ltd. (Shanghai, China), into the tail vein of ApoE-/- mice, according to a previous study [44]. A Lv vector expressing green fluorescence protein only was used as the control. Mice were randomly divided into five experimental groups (n = 8/group): (I) the C57 mouse group; (II) ApoE-/- mouse group; (III) ApoE-/- mouse + AsC group; (IV) ApoE-/- mouse + BECN1 RNAi group; and (V) ApoE-/- mouse + AsC + BECN1 RNAi group. One month after the injection of Lv, the mice were subjected to either AsC (20 mg·kg^-1^·day^-1^, i.g.) or normal saline for 4 weeks as described above (Figure 2A). All mice were fed with a high fat diet (HFD, 0.3% cholesterol and 20% pork fat) for 4 weeks. AsC was dissolved in normal saline.

### Serum lipids

At the end of the experiment, all mice were fasted overnight and their sera were acquired from the inner canthus. Then, the heart and aortas were separated immediately, and the aortas were stored at -80°C. Serum lipid levels were determined based on commercial kits using a Beckman AU480 biochemical autoanalyzer (Fullerton, CA, USA).

### Histological and immunohistochemical assays

Serial cryo-sections (6 μm thick) of the aortic root were stained with oil red O. Sirt1, Cd86, Arg1 expression in the aortic root was detected by immunofluorescence. Briefly, the aortic sections were incubated with primary antibody (1:50) overnight at 4°C. After rinsing, the sections were incubated for 1 h with the secondary antibody at room temperature and then incubated with 0.5 g/L DAPI containing antifluorescence quenching agent for 5 min. Images were obtained with a the Tissue FAXS microscope (Tissue FAX plus; Tissue Gnostics, Vienna, Austria) and analyzed based on our previous method [22].

### Cell culture and treatment

RAW264.7 macrophages were obtained from the National Infrastructure of Cell Line Resource (Beijing, China), and cultured in DMEM basic medium supplemented with 10% (v/v) FBS and 1% penicillin-streptomycin. Macrophages were maintained in a 5% CO2 incubator at 37°C. AsC was dissolved in DMSO to generate a solution and diluted with culture medium. Then, the cells were seeded in various plates, pretreated with AsC, and exposed to ox-LDL (80 μg·mL^-1^) for 24 h.

### Oil red O staining

Macrophages were pretreated with AsC (20 μM) for 12 h and then incubated with ox-LDL for 24 h. The cells were washed three times with PBS and fixed with 4% (w/v) paraformaldehyde for 10 min at room temperature. After that, the cells were stained with filtered oil red O solution (30 min, 60°C) and observed under a microscope (Olympus, Tokyo, Japan). Then, the absorbance was measured at 358 nm by a Tecan Infinite M1000 Microplate Reader (Tecan, Männedorf, Switzerland).

### Transmission electron microscopy (TEM)

After all treatments, the cells were collected and fixed in 2.5% glutaraldehyde (TAAB, Berkshire, England) in 0.1 M sodium phosphate buffer (pH 7.4) overnight. The cells were washed in the same buffer 3 times and postfixed in 1% osmic acid at 4°C for 2 h and then dehydrated and embedded in epoxypropane according to a standard procedure. Ultrathin sections were stained with uranyl acetate and lead citrate and observed under a JEOL JEM1230 (JEOL Ltd., Tokyo, Japan).

### Flow cytometry

Autophagosome formation in macrophages was investigated using a CytoID Autophagy Detection Kit (Enzo Life Sciences, NY, USA) according to the manufacturer’s instructions. The CytoID fluorescent reagents specifically evaluated autophagic vacuoles formed during autophagy. Briefly, macrophages were obtained and washed twice in PBS. The cells were resuspended in 0.5 mL of freshly diluted CytoID reagents and incubated at 37 °C for 30 min. The cells were washed and the CytoID fluorescence of the cells was immediately analyzed by flow cytometry (BD Biosciences, NJ, USA). The percentage of cells with CytoID staining was used to represent the formation of autophagosomes.

Macrophages were surface stained with anti-Cd36 and anti-Mrc1 antibodies and then analyzed by flow cytometry. Briefly, macrophages were collected and stained with anti-Cd36 and anti-Mrc1 antibodies for 20 min at 37°C, washed with PBS and analyzed using flow cytometry. The fluorescence intensity was statistically compared with model group.

### Arginase assay

Arginase activity was measured based on a previous study [49]. Briefly, macrophages were collected and lysed with 0.1% Triton X-100 (containing protease and phosphatase inhibitors). Then, the cell lysate was incubated with arginase activation solution (5 mM MnCl2 in 25 mM Tris HCl [pH 7.4]) for 10 min at 56°C. Subsequently, the mixture was added to the arginase substrate solution (0.5 M L-arginine in water [pH 9.7]) and incubated at 37°C for 1 h. The reaction was terminated by the addition of an acid mixture (H2SO4, H3PO4, and water at a ratio of 1:3:7), followed by the addition of a-isonitrosopropiophenone (9% w/v), which was heated to 100°C for 45 min. The OD value was measured at 540 nm on a microplate reader (Tecan, Switzerland).

### Immunofluorescence

Cell immunofluorescence staining was performed using rat anti-LC3II, anti-Mrc1, anti-ATG5 antibodies as well as rat IgG (H+L) F(ab')2 fragment. DAPI was used to visualize the nuclei. The cells were observed by the ImageXpress® Micro system (Molecular Device, CA, USA) and analyzed by MetaXpress Software according to our previous study [36].

### Quantitative real-time PCR

The RAW264.7 cells were pretreated with AsC (20 μM) 12 h before exposure to ox-LDL for 24 h. Total RNA was extracted using TRIzol (Invitrogen, Carlsbad, CA, United States). The isolated RNA was reverse transcribed into cDNA using the GoScriptTM Reverse Transcription System (Promega). Then, amplification was carried out using real-time RT-PCR with the Power SYBR Premix Ex TaqTM II (TaKaRa Biotechnology, Dalian, China) in an iQ5 Real-time PCR detection system with analysis software (Bio-Rad, Santa Rosa, CA, United States). Primers (Table 1) were designed using premier primer Software 6.0 (Canadian Premier Life Insurance Company, ON, Canada). The 2^-ΔCT^ method was used to analyze the results according to our previous research [24].

**Table 1:** Primers used for quantitative real-time PCR.

**Gene**	**Primer sequence (5' to 3')**	**Product (bp)**
GAPDH	F: CTGCGGCATCCACGAAACT	126
	R: AGGGCCGTGATCTCCTTCTG	
IL-1β	F: TGCCACCTTTTGACAGTGATGA	135
	R: TGTGCTGCTGCGAGATTTGA	
iNOS	F: CTGCAGCACTTGGATCAGGAACCTG	311
	R: GGAGTAGCCTGTGTGCACCTGGAA	
CD206	F: TGCTACTGAACCTCCTCAACTGC	121
	R: AGCCTGACCCCAACTTCTCGT	
Arg-1	F: TGCATATCTGCCAAAGACATCG	137
	R: TCCATCACCTTGCCAATCCC	

### Cellular thermal shift assay (CETSA)

CETSA was performed according to a previous study [26]. Briefly, the cells were collected and heated individually at different temperatures (42, 46, 50, 54, 58, and 62°C) for 3 min and then cooled for 3 min at room temperature. Then, the samples were centrifuged, and the obtained cells were analyzed by western blotting.

### Drug affinity responsive target stability (DARTS) assay

Briefly, total cell protein was isolated using NP40 lysis buffer. The lysate was equally distributed into seven groups and treated with different concentrations of DMSO as a control or AsC (0, 5, 10, 20, 50 and 100 μM) separately for 1 h at room temperature. Then, pronase (25 μg·mL^-1^) was added to the lysates for a further an additional 30 min at 37°C. The reactions were stopped by adding SDS-PAGE loading buffer, and the samples were analyzed via western blotting.

### Molecular docking

The binding poses of AsC in the active site of Sirt1 (PDB code: 4ZZJ) were analyzed using the docking program Lib Dock according to our previous method [21]. The binding affinities (LibDockScore) in Discovery Studio 4.5 were used to evaluate the interactions between AsC and Sirt1.

### siRNA assay

siRNAs targeting BECN1 and Sirt1 were purchased from Santa Cruz Biotechnology along with control siRNA and siRNA transfection reagent, according to our previous report [36]. Briefly, 80% confluent cells were transiently transfected with 100 nM siRNA per dish for 7 h according to the manufacturer's method. Then, the cells were switched to DMEM complete medium and incubated for an additional 24 h. Where indicated, macrophages were treated with AsC (20 μM) for 12 h and then exposed to ox-LDL for another 24 h. The knockdown efficiency of the target proteins was measured by western blotting.

### Western blot analysis

Macrophages were harvested and lysed with cell lysis buffer containing 0.1 mM dithiothreitol and proteinase inhibitor cocktail. Protein concentration was detected using a Bio-Rad DC protein determination kit. A western blot assay was then performed, and immunoblotting was developed using an ECL kit. Band intensities were analyzed using Gel Pro software (Media Cybernetics, Rockville, MD, United States).

### Statistical analysis

All analyses were performed with GraphPad Prism 6.0 software (San Diego, CA, USA). The data are presented as the mean ± S.D. Multigroup comparisons were analyzed by one-way analysis of variance (ANOVA) followed by Tukey's post hoc test. Comparisons between two groups were performed by use of Student's unpaired t-test. Values of *P* < 0.05 were considered to indicate statistical significance. The data and statistical analysis complied with the recommendations on experimental design and analysis in pharmacology (Curtis et al., 2018).

## Supplementary Material

Supplementary Figures

Supplementary Methods and References
