# Correction for: Araloside C attenuates atherosclerosis by modulating macrophage polarization via Sirt1-mediated autophagy

**DOI:** 10.18632/aging.206100

**Published:** 2024-08-31

**Authors:** Yun Luo, Shan Lu, Ye Gao, Ke Yang, Daoshun Wu, Xudong Xu, Guibo Sun, Xiaobo Sun

**Affiliations:** 1 Institute of Medicinal Plant Development, Peking Union Medical College and Chinese Academy of Medical Sciences, Beijing 100193, China; 2 Beijing Key Laboratory of Innovative Drug Discovery of Traditional Chinese Medicine (Natural Medicine) and Translational Medicine, Beijing 100193, China; 3 Key Laboratory of Bioactive Substances and Resource Utilization of Chinese Herbal Medicine, Ministry of Education, Beijing 100193, China; 4 Key Laboratory of Efficacy Evaluation of Chinese Medicine Against Glyeolipid Metabolism Disorder Disease, State Administration of Traditional Chinese Medicine, Beijing 100193, China; 5 Key Laboratory of New Drug Discovery Based on Classic Chinese Medicine Prescription, Chinese Academy of Medical Sciences, Beijing 100193, China; 6 College of Pharmacy, Harbin University of Commerce, Harbin 150076, Heilongjiang, China; 7 Collaborative Innovation Center of Yangtze River Delta Region Green Pharmaceuticals, Zhejiang University of Technology, Hangzhou 310014, Zhejiang, China

**Keywords:** atherosclerosis, macrophage polarization, autophagy, Sirt1, Araloside C

**This article has been corrected:** The authors recently found that three images in **Figures 4** and **5** contain the duplications. The authors confirmed that they inadvertently used the same representative micropgraph of autophagosomes from the ox-LDL+AsC+3-MA group in **Figure 4A** for the control group. In **Figure 5D**, the image of oil red O-stained ox-LDL-treated RAW264.7 cells is the same image used for ox-LDL+AsC+3-MA group. In addition, the image from the ox-LDL+3-MA group in **Figure 5D** was a duplication of the ox-LDL group image in **Figure 2F**. The authors provided original pictures for all groups and confirmed that other results were not compromised by these mistakes. They replaced the incorrect images with correct ones from the original experiments and stated that this correction has no impact on the experimental outcome or conclusions. The authors would like to apologize for any inconvenience caused.

The corrected version of **Figures 4** and **5** are provided below.

**Figure 4:**
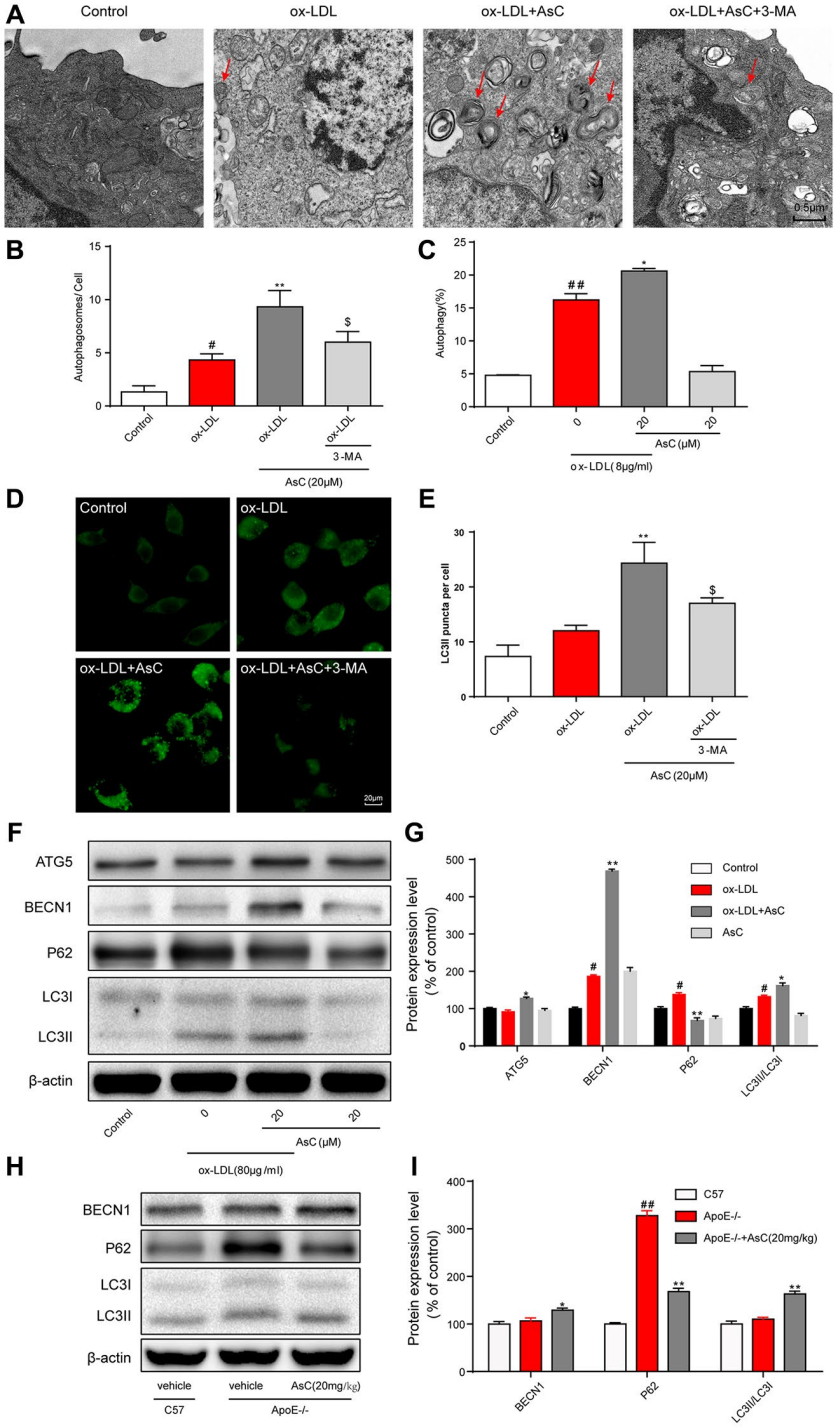
**AsC induced macrophage autophagy. RAW264.7 cells were pretreated with 3-MA (5 mM) for 2 h, treated with AsC (20 μM) for 12 h, and then exposed to ox-LDL for another 24 h.** (**A**) Representative photographs of autophagosomes (red arrows) examined using a JEOL JEM1230 electron microscope. (**B**) Statistical results of autophagosomes. (**C**) Summarized data showing the percentage of cells that were positive for CytoID fluorescence, as detected by flow cytometry analysis. (**D**) Representative photographs of LC3II staining. (**E**) Statistical results of LC3II-positive cells. (**F**) Representative photographs of ATG5, BECN1, P62, LC3 and β-actin expression in ox-LDL-treated macrophages, as evaluated by western blot analysis. (**G**) Statistical results of ATG5, BECN1, P62, LC3II/LC3I expression levels compared with those in the control group. (**H**) Representative photographs of BECN1, P62, LC3 and β-actin expression in aortic lysates. (**I**) Statistical results of BECN1, P62, LC3II/LC3I expression levels compared to those in the control group. The data are presented as the means ± SDs (*n* = 5). ^#^*P* < 0.05, ^##^*P* < 0.01 vs. the control group, ^*^P < 0.05, ^**^P < 0.01 vs. the model group; ^$^*P* < 0.05 vs. the ox-LDL and AsC group.

**Figure 5:**
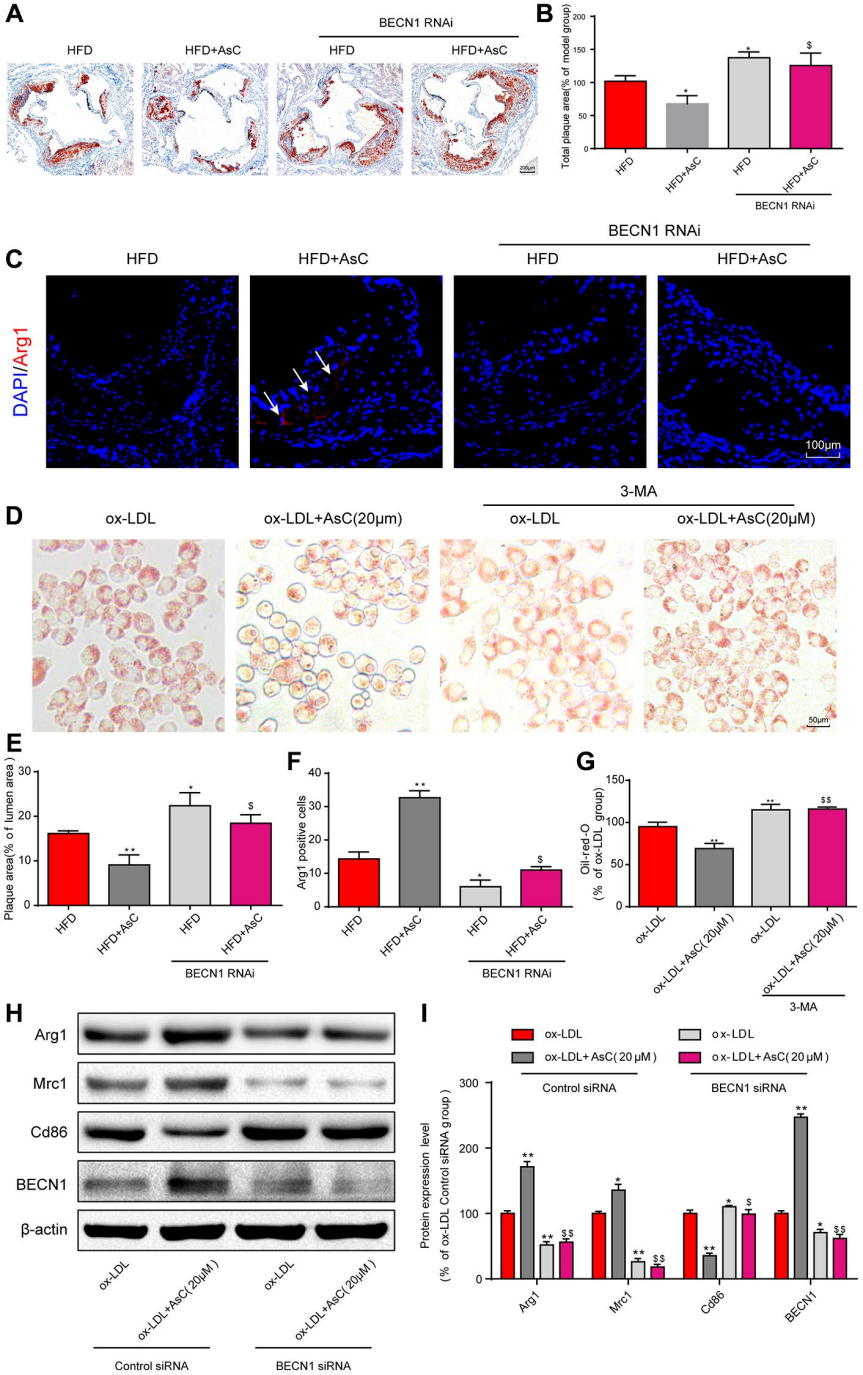
**Autophagy inhibition abolished AsC-mediated antiatherosclerotic effects and macrophage polarization.** All mice were fed a HFD in the presence or absence of AsC (20 mg·kg^−1^·day^−1^, i.g.) for 4 weeks. In the* in vitro* assay, RAW264.7 cells were pretreated with 3-MA (5 mM) for 2 h, treated with AsC (20 μM) for 12 h, and then exposed to ox-LDL for another 24 h. (**A**) Representative images of oil red O staining of the aortic root. (**B**) Quantification of the total plaque area. (**C**) Dual immunofluorescence staining forArg1 (red) and DAPI (blue) in lesions in the aortic root. (**D**) Representative images of oil red O staining of ox-LDL-treated RAW264.7 cells. (**E**) The percentage of plaque area relative to lumen area. (**F**) Quantification of relative fluorescence intensity. (**G**) Quantification of oil red O staining, as detected by a microplate reader. (**H**) Representative photographs of Arg1, Mrc1, Cd86 and BECN1 expression, as evaluated by western blot analysis. (**I**) Statistical results of Mrc1, Cd86 and Arg1 expression levels compared with those in the ox-LDL-treated group. The data are presented as the means ± SDs (*n* = 5). ^*^P < 0.05, ^**^P < 0.01 vs. the model group; ^$^*P* < 0.05, ^$$^*P* < 0.01 vs. the ox-LDL and AsC group.

