# Supplementary material for: Araloside C attenuates atherosclerosis by modulating macrophage polarization via Sirt1-mediated autophagy
Source: Aging (Albany NY). 2020 Jan 27;12(2):1704–24. doi: 10.18632/aging.102708 (PMC7053643; doi:10.18632/aging.102708)
Supplement: Supplementary Figures [file aging-12-102708-s002..pdf]

SUPPLEMENTARY FIGURES

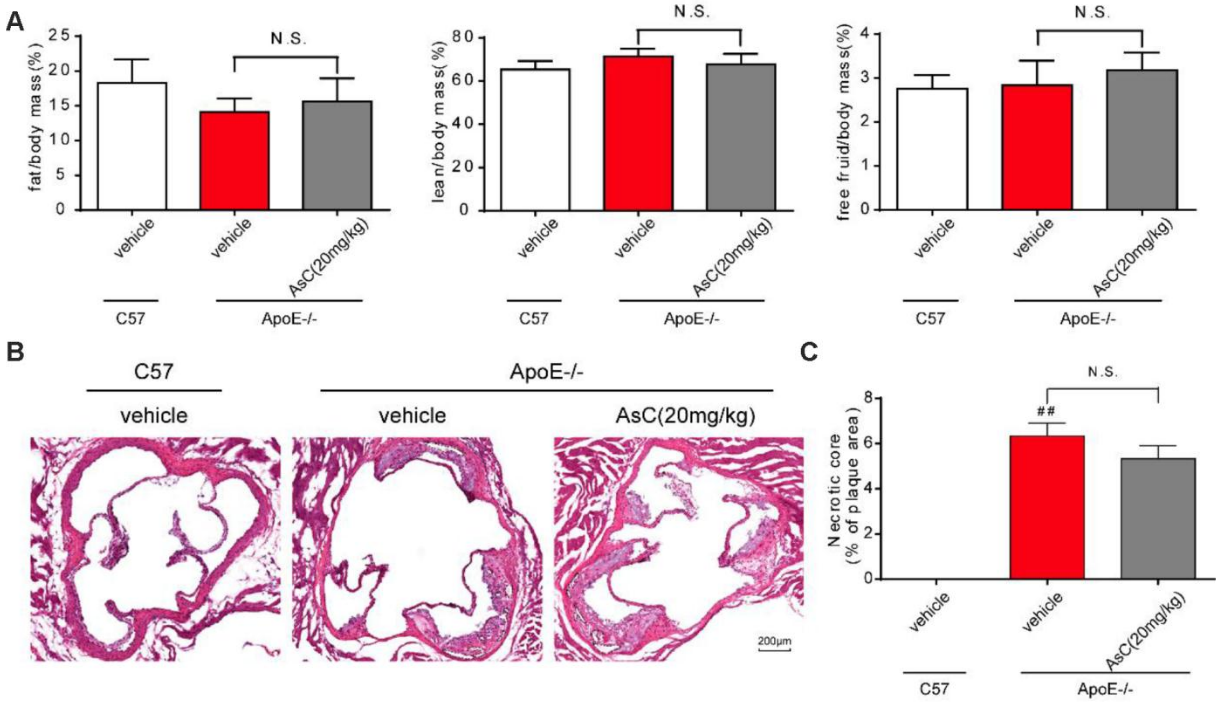

Supplemetary Figure 1. The effects of AsC on body component and necrosis area in HFD-treated mice. (**A**) The fat, lean and free fruid level in all mice. (**B**) HE staining of aortic root in all mice. (**C**) The statistic results of nercrotic core described in (**B**). Data are presented as means ± SD (n = 5). $^{\#\#}P < 0.01$ *vs*. the Control group; N.S. means no significance.

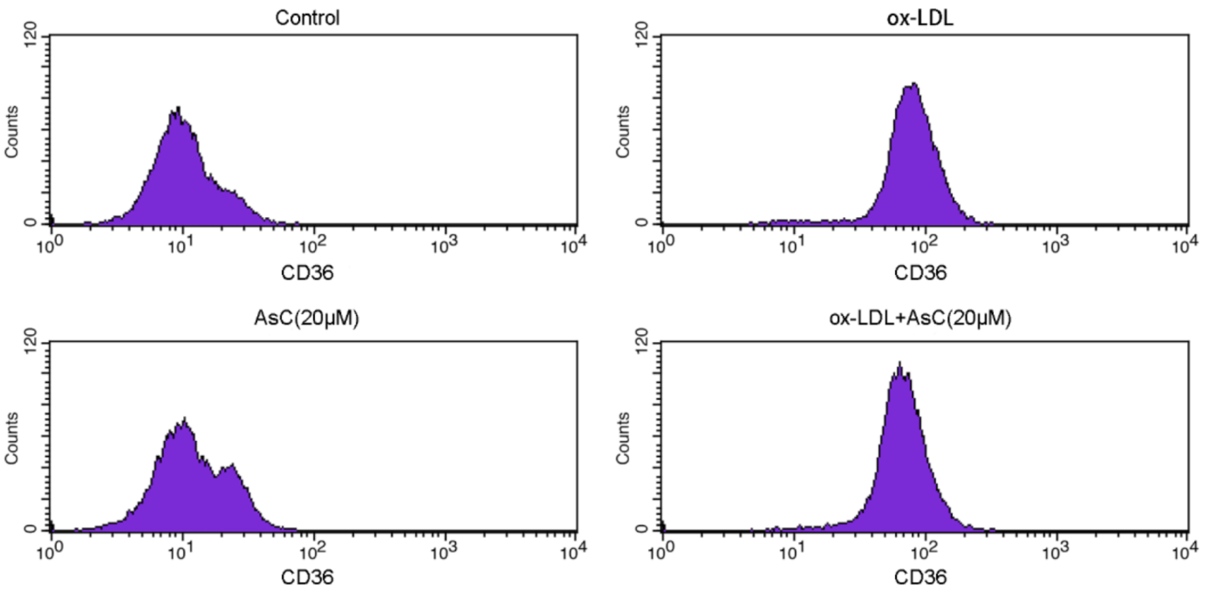

Supplementary Figure 2. The representive results of Cd36 detected by flow cytometry.

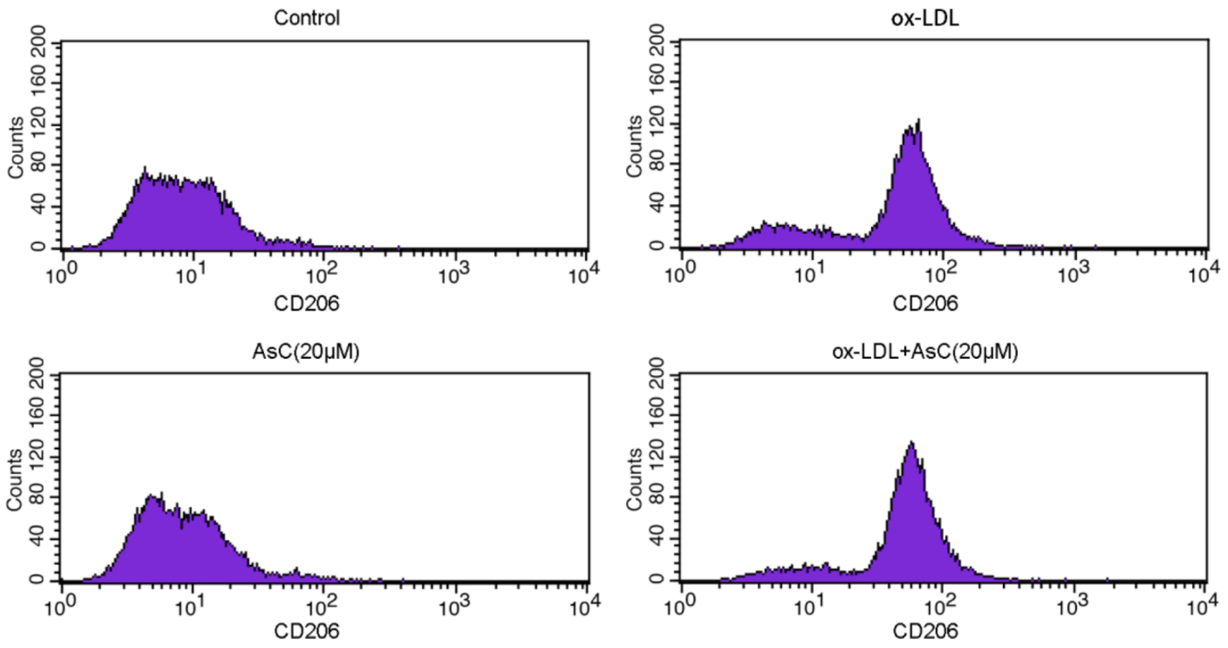

Supplementary Figure 3. The representive results of Mrc1 detected by flow cytometry.
