# Supplementary material for: Araloside C attenuates atherosclerosis by modulating macrophage polarization via Sirt1-mediated autophagy
Source: Aging (Albany NY). 2020 Jan 27;12(2):1704–24. doi: 10.18632/aging.102708 (PMC7053643; doi:10.18632/aging.102708)
Supplement: Supplementary Methods and References [file aging-12-102708-s001..pdf]

Supplementary Methods

Body composition and body weight determination

Body composition was measured using the Bruker LF65 II "Minispec" body composition analyzer (Bruker Optics, Billerica, MA) as previously reported (Gordon et al., 2016). Briefly, a mouse was placed in an acrylic cylinder (48-mm diameter) and was loosely restrained in the cylinder by pushing a plunger to maintain the mouse inside the cylinder based on the size of the mouse. The cylinder was then positioned inside the bore of the magnet. The measurements of fat, lean, and fluid were recorded in 2 min. And the % fat, lean and fluid relative to body weight were calculated.

HE staining

Frozen sections of the aortic root were stained with oil red O according to our previous method (Luo et al., 2015).

Supplementary References

1. Gordon CJ, Phillips PM, Johnstone AF. A noninvasive method to study regulation of extracellular fluid volume in rats using nuclear magnetic resonance. Am J Physiol Renal Physiol. 2016; 310:F426–31. https://doi.org/10.1152/ajprenal.00405.2015. PMID:26697983
2. Luo Y, Dong X, Yu Y, Sun G, Sun X. Total aralosides of aralia elata (Miq) seem (TASAES) ameliorate nonalcoholic steatohepatitis by modulating IRE1α-mediated JNK and NF-κB pathways in ApoE-/- mice. J Ethnopharmacol. 2015; 163:241–50. https://doi.org/10.1016/j.jep.2015.01.017. PMID:25655997
